# Ozone

**Published:** 1869-11

**Authors:** 


### *Ozone.*

Have the physicians of this section noticed the remarkable abundance of ozone in the atmosphere during the past six weeks? The unusual prevalence of catarrhal disorders directed our own attention to the subject, and almost daily observations have shown a remarkable amount of this element present. The test employed was the simple one of Polli's. These autumn days have thus been proved, as Schönbein felicitously termed them, *blue* days. Our own experience has further shown that there has been from, perhaps, the same cause, an unusual prevalence of catarrhal diarrhœas, as well as affections of the respiratory mucus membranes.

